# Effect of phytol in forage on phytanic acid content in cow’s milk

**DOI:** 10.5713/ab.21.0086

**Published:** 2021-06-23

**Authors:** Renlong Lv, Mabrouk Elsabagh, Taketo Obitsu, Toshihisa Sugino, Yuzo Kurokawa

**Affiliations:** 1 Tropical Crops Genetic Resources Institute, Chinese Academy of Tropical Agricultural Sciences, Haikou 571101, China; 2 Department of Animal Production and Technology, Faculty of Agricultural Sciences and Technologies, Nigde University, Nigde 51240, Turkey; 3 Department of Nutrition and Clinical Nutrition, Faculty of Veterinary Medicine, Kafrelsheikh University, Kafr El-Sheikh 33516, Egypt; 4 Graduate School of Integrated Sciences for Life, Hiroshima University, Higashi-Hiroshima 739-8528, Japan

**Keywords:** Milk, Phytanic Acid, Phytol, Silage

## Abstract

**Objective:**

Bioactive compounds in ruminant products are related to functional compounds in their diets. Therefore, this study aimed to explore the effect of forage sources, Italian ryegrass (IR) silage vs corn silage (CS) in the total mixed ration (TMR), on milk production, milk composition, and phytanic acid content in milk, as well as on the extent of conversion of dietary phytol to milk phytanic acid.

**Methods:**

Phytanic acid content in milk was investigated for cows fed a TMR containing either IR silage or CS using 17 cows over three periods of 21 days each. In periods 1 and 3, cows were fed CS-based TMR (30% CS), while in period 2, cows were fed IR silage-based TMR (20% IR silage and10% CS).

**Results:**

The results showed that there were no differences in fat, protein, lactose, solids-not-fat, somatic cell count, and fatty acid composition of milk among the three experimental periods. There were no differences in the plasma concentration of glucose, triglycerides, total cholesterol, and nonesterified fatty acids among the three experimental periods, while the blood urea nitrogen was higher (p<0.05) in period 2. The milk phytanic acid content was higher (p<0.05) in period 2 (13.9 mg/kg) compared with periods 1 (9.30 mg/kg) and 3 (8.80 mg/kg). Also, the phytanic acid content in the feces was higher (p<0.05) in period 2 (1.65 mg/kg dry matter [DM]) compared with period 1 (1.15 mg/kg DM), and 3 (1.17 mg/kg DM). Although the phytol contents in feces did not differ among the three feeding periods, the conversion ratio from dietary phytol to milk phytanic acid was estimated to be only 2.6%.

**Conclusion:**

Phytanic acid content in cow’s milk increases with increasing phytol content in diets. However, phytol might not be completely metabolized in the rumen and phytanic acid, in turn, might not be completely recovered into cow’s milk. The change of phytanic acid content in milk may be positively correlated with the change of phytol in the diet within a short time.

## INTRODUCTION

Ruminant products (milk and meat) are known to contain bioactive compounds that contribute to human health [1]. Among these compounds which have beneficial properties for human health that contribute to metabolic syndrome prevention, include phytanic acid (a fatty acid) derived from the phytol moiety of chlorophyll [2,3]. The phytanic acid content in milk has been reported to vary with forages level, species, and conservation methods depending on the phytol content of feed; it increases with increasing fresh forage [4,5], silage instead of hay [6], or red clover rather than grass silage [7] intake. The determinant factors of phytanic acid content in ruminant products are the chlorophyll content in forage and the amount of phytol liberated in the rumen. Therefore, effective utilization of chlorophyll and phytol in forage could improve the additional value of ruminant products and could have positive effects on the health of cows. Despite some papers reported the phytanic acid content in milk products [4] and the change of chlorophyll and phytol in herbage during the preservative process [8,9], no reports confirmed the relationship between the phytol intake and phytanic acid in milk.

Since more attention being paid to organic farming, making flexible use of forage is an important strategy for agricultural development in the future, and exploring the potential value of forage is a necessary study. Italian ryegrass (IR; *Lolium multiflorum* Lam.), is one of the most important forage crops. IR is now widely distributed through temperate areas of the world and generally regarded as the basis of grassland improvement because of its high nutritional value, digestibility, and well ensiling characteristics [10]. IR is also used as a major silage crop in Japan and has been widely used for silage making [11]. Whole crop corn silage (CS) contains leaves, steams, grains and cobs so that chlorophyll or phytol content would be diluted with the non-leaf part of the plant. Thus, the phytol content is expected to be higher in IR silage compared with CS. In dairy production systems, total mixed rations (TMR) containing forages, grains, protein feeds, minerals, vitamins, and feed additives are used to satisfy the nutrient requirement of cows [12]. The phytanic acid content in milk of cows fed TMR containing IR silage is expected to be higher than that of cows fed TMR containing CS, due to the difference in the phytol content between IR silage and CS.

Therefore, this study aimed to explore the effect of forage sources, IR silage vs CS in the TMR on milk production, milk composition, and phytanic acid content in milk, and the extent of conversion of dietary phytol to milk phytanic acid.

## MATERIALS AND METHODS

### Experimental design and animals

All animal procedures were managed according to the guidelines of the Animal Care and Use Committee of Hiroshima University. A total of 17 Holstein cows (8 primiparous and 9 multiparous cows) averaging (mean±standard deviation) 1.9±1.2 parity, 213±97 days in milk, 732±65 kg of body weight, and 31.3±8.8 kg/d milk production, were used in the experiment consisting of three 21-d periods at Hiroshima University Farm. Cows were raised in the cowshed installing an automatic milking system (Astronaut A3 next, Lely, the Netherland) and the roughage intake control system (Insentec, Drachten, the Netherland). Cows were supplied with a concentrate diet with an automatic feeder in the automatic milking system. The CS-based TMR was fed during the first and third periods (period 1 and period 3), and the IR silage-based TMR was fed during the second period (period 2). The ingredients and chemical composition of the TMR are shown in Table 1. The milk samples were collected at the last 2 days of each period and preserved at −30°C for the later determination of the milk components. The blood of the caudal artery was collected at 13:00 on the last day of each period. Then, plasma was collected after centrifugation at 4°C for 10 min and preserved at −30°C for further analyses. Feed samples were collected over the last 3 days of each experimental period and freeze-dried for later analysis. Spot fecal samples were collected from 4 cows over the last 3 days of each experimental period. The feces of each cow were collected immediately after defecation in the morning (8:30 to 9:30), afternoon (16:30 to 17:30), and evening (00:30 to 1:30), then they were mixed completely and freeze-dried for later analysis.

### Chemical analysis

Feed samples were analyzed for dry matter (DM), crude ash, crude protein (CP), and ether extract by the methods of AOAC [13], and the neutral detergent fiber (NDF) was determined according to Van Soest et al [14].

Milk samples were measured for fat, protein, lactose, and solids-not-fat (SNF) by an infrared analyzer (Lactoscope Filter C4+, Delta Instruments, Drachten, and the Netherlands). Somatic cell count (SCC) was analyzed by milk somatic cell counter (NucleoCounter, SCC-100.chemometec. Allerod, Denmark). Plasma samples were analyzed for glucose, non-esterified fatty acid (NEFA), triglyceride (TG), total cholesterol (T-CHO), and blood urea nitrogen (BUN) using an automated biochemical analyzer (AU 480; Beckman Coulter, Brea, CA, USA).

The fatty acid content including phytanic acid in milk samples was determined by gas chromatography/mass spectrometry (GC-MS) (QP2010, Ultra, Shimadzu, Kyoto, Japan) after acid methylation of lipid extracts according to the methyl esterification method for fatty acid analyses of feeds [15]. Briefly, 1 mL of milk was mixed with 0.2 mL 28% ammonia solution and 0.8 mL 96% ethanol. Then, 0.2 mL of methyl tridecanoate (2.5 mg/ml hexane) was added as an internal standard. 1 mL 0.025% butylated hydroxytoluene diethyl ether and One mL hexane were added and mixed well. The supernatant after centrifugation at 1,710 *g* for 5 min was collected. The extraction process was repeated and the combined supernatant was dried under N_2_ gas stream. Then, 3 mL 0.78 N hydrochloric acid (HCI) in methyl alcohol and 2 mL chloroform were added to the tube, and the tube was heated for 2.5 h at 65°C. Then 5 mL solution of 6% K_2_CO_3_ and 3 mL hexane was mixed completely. After centrifugation, the supernatant was loaded onto a column containing 0.5 g florisil, and then the column was eluated with 5 mL hexane with diethyl ether (95:5 V/V). The eluate was dried at 40°C under a constant stream of N_2_ gas and redissolved in 1 mL hexane for gas chromatography-mass spectrometer (QP2010, Ultra, Shimadzu, Japan) equipped with SP-2560 (100 m×0.25 mm, film thickness 0.2 μm; Supelco, Bellefonte, PA, USA). Helium was used as a carrier gas. The column pressure was set at 170 kPa. The initial temperature of the column oven at 70°C for 3 min was raised to 130°C by 11°C/min, then to 160°C by 1°C/min, finally raised to 220°C by 3°C/min. The split ratio was 60.0. The injection volume was 1 μL. For the mass spectrometer, ion source temperature and interface temperature were set at 200°C and 240°C respectively. Selected ion mode was used to measure the relative intensity of 101 and 87 m/z fragments as target ions of methyl ester of phytanic acid and nonadecanoic acid, respectively. Phytol in TMR and feces was analyzed following the methods of Liljenberg and Odham [16] and Takeda et al [17]; detailed analytical procedures are previously described [8]. Phytanic acid in feces was also determined by GC-MS (QP2010, Ultra, Shimadzu, Japan). Tridecanoic acid (0.25 mg/mL, 1 mL) was used as an internal standard solution. In a screw-capped tube, the internal standard solution (1 mL) was added to 0.1 g freeze-dried feces samples and then dried under N_2_ stream at 40°C. And the following treatments were the same as the above methods.

### Statistical analysis

Statistical analysis was performed using the general linear model procedure of SAS [18]. The data were analyzed as a complete blocked design. Tukey’s test was used to identify the differences of means (p<0.05) among experimental periods.

## RESULTS

Chemical compositions of the TMR had no differences in DM, CP, and NDF among the periods (Table 1). The phytol content in the TMR at period 2 (0.784 g/kg DM) was higher (p<0.05) than that at periods 1 (0.483 g/kg DM) and 3 (0.517 g/kg DM). The DMI of TMR and concentrate were 19.5, 19.7, 20.1 kg/d and 4.9, 4.8, 4.6 kg/d, for periods 1, 2, and 3, respectively, but there were no significant differences among the three experimental periods (Table 2). The daily milk yield averaged 28.4 kg/d which was similar among the three experimental periods. There were no differences in fat, protein, lactose, SNF, and SCC content in milk among the three experimental periods (Table 2). There were no differences in fatty acid composition in milk among the three experimental periods (Table 3). There were no differences in the plasma concentration of glucose, TG, T-CHO, and NEFA among the three experimental periods, while the BUN was higher (p<0.05) at period 2 (Table 4). The milk phytanic acid content was higher (p<0.05) at period 2 compared with that of periods 1 and 3 (Table 5). Also, the phytanic acid content in feces was higher (p<0.05) in period 2 compared with that of periods 1 and 3. Phytol contents in feces had no differences among the three feeding periods (Table 5).

## DISCUSSION

This experiment aimed to explore the conversion ratio of dietary phytol to milk phytanic acid in dairy cows fed TMR with different phytol contents. The absence of differences in DMI, milk yields, and milk composition among the periods indicates that the difference of silage source in TMR did not affect milk production performance due to the similar energy intake. The results are consistent with other reports [19,20].

The phytanic acid content in milk was higher for period 2. Schröder et al [4] reported that phytanic acid content in milk was between 0.021 and 0.2 mg/g milk. However, the phytanic acid content in this experiment was lower (9.3, 13.9, and 8.8 mg/kg for periods 1, 2, and 3, respectively) than that of their report, presumably due to the low phytol content in the TMR used in this study. In our experiment, silage and hay (Oats hay and alfalfa hay) accounted for 50% of TMR, while Schröder et al [5] used diets containing 86% of silage and hay. In addition, the feeding conditions and diets of cows were also important effective factors for milk quality [21]. The phytol intakes from TMR were calculated to be 9.5, 15.5, and 10.4 g/d for periods 1, 2, and 3 respectively. The phytol intake during period 2 was higher (p<0.05) compared with other periods. Also, the total phytanic acid secretion into the milk was calculated to be 0.27, 0.42, and 0.25 g/d for periods 1, 2, and 3 respectively. Based on the calculation, the conversion ratio of dietary phytol to milk phytanic acid was estimated to be only 2.6%. The phytanic acid content in feces was 1.2, 1.6, and 1.2 mg/kg for periods 1, 2, and 3, respectively. These results indicate that not all the phytanic acid produced in the rumen could be utilized by cows, and part of it is excreted into the feces. A slightly higher phytanic acid content in the feces was observed for period 2. This higher excretion was also affected by phytol intake. Because the total digestible nutrients content of TMR diets was about 70% for the three periods, DM digestibility of the TMR can be assumed to be 70%. Based on this assumption, fecal excretion of phytanic acid was estimated to be very small, only 0.07% of phytol intake. Thus, most of the dietary phytol was not recovered as phytanic acid in milk nor feces. This low appearance of phytanic acid was presumably owing to the low phytanic acid production in the rumen. In a previous study, the phytanic acid conversion ratio of IR silage with different phytol contents in the rumen was addressed; the conversion ratio of phytanic acid was only 15% to 36% and most phytol remained in the rumen [22], which was likely related to rumen microbial composition, diet composition, raising condition and feeding methods, etc. Previous studies demonstrated that dietary composition would affect the species and concentration of rumen microorganisms and consequently, affect phytanic acid production [23]. Therefore, under the condition of this study, most phytanic acid in the rumen might be utilized for purposes other than the milk component. Further studies on the factors affecting phytanic acid production in the rumen are necessary. In addition, phytol was found in feces, and there were no differences in the phytol content among the three periods. Using the above assumption, the fecal excretion of phytol was estimated to be 3.3, 2.8, and 2.9 g/d for periods 1, 2, and 3 respectively. This phytol excretion accounted for 35% (period-1), 19% (period-2), and 30% (period-3) of dietary phytol, respectively. Although the phytol intake of cows fed IR silage-based TMR was higher, the excreted ratio was lower for the cows fed IR. Compared with CS-based TMR, the apparent use of phytol in the total digestive tract seems to be higher for IR silage-based TMR. In this experiment, there were no differences in fatty acid profile in milk. The results are consistent with other reports [24]. Herbage is usually rich in C18:3 fatty acid [25] so that C18:3 in milk is one of the important fatty acid markers in some organic milk systems. The relationship between the C18:3 in milk fat and phytanic acid production was observed in some reports and showed that C18:3 was 3 times higher than phytanic acid [4]. However, no differences in C18:3 were found among the three feeding periods in this experiment. Although milk C18:3 could reflect the fatty acid composition in diets, it could not be regarded as a marker for phytanic acid content in milk.

The concentration of BUN in period 2 was higher than that in periods 1 and 3. The different BUN concentrations among the experimental periods might be due to a balance between hepatic production and output (urinary excretion and recycling) of urea-N [26]. BUN is affected by protein and energy consumed by animals and the breakdown of muscle protein [27]. In addition, it was reported that there was a positive correlation between BUN and ruminal ammonia [28,29]. Ruminal ammonia was utilized by rumen microorganisms [30]. Different components of diets or protein would affect ruminal ammonia content [31]. In this experiment, thus, the IR silage-based TMR would have a higher degradable N compared with the CS-based TMR.

In conclusion, the phytanic acid content in milk was higher for cows fed the IR silage-based TMR compared with the CS-based TMR. However, the conversion ratio of dietary phytol into milk phytanic acid was estimated to be only 2.6%. There were no differences in milk yield and milk composition contents between cows fed the IR silage-based TMR and CS-based TMR. Further studies are warranted on the factors affecting phytanic acid production in the rumen.

## Figures and Tables

**Table 1:** Ingredients and composition of total mixed rations for cows at each experimental period

Item	Period -1^1)^	Period -2^1)^	Period -3^1)^	SEM
Ingredient (% of DM)				
Italian ryegrass silage	0	20.4	0	-
Corn silage	30.2	9.5	30.2	-
Oats hay	9.9	7.1	9.9	-
Alfafa hay	11.1	12.7	11.1	-
Beet pulp	6.6	6.9	6.6	-
Concentrate mixture	40.2	41	40.2	-
CaCO_3_	0.8	1	0.8	-
Vitamin	0.9	1	0.9	-
NaCl	0.4	0.4	0.4	-
Composition (% of DM)				
Dry matter (% of FM)	44.7±0.96	46.0±2.20	47.1±0.43	5.96
Crude protein	12.6±0.21	13.0±0.40	13.5±0.59	0.56
NDFom	40.3±0.09	42.3±0.93	41.0±0.89	1.66
Ether extract	3.15±0.09	3.09±0.06	3.1±0.11	0.03
Ca	0.85	0.83	0.8	-
P	0.38	0.37	0.36	-
TDN	66.9	68.7	69.3	-
Phytol (g/kg DM)	0.483±0.01^b^	0.784±0.08^a^	0.517±0.02^b^	0.007

SEM, standard error of means; DM, dry matter; FM, fresh matter; NDFom, neutral detergent fiber exclusive of residual ash; TDN, total digestible nutrients.

1) Cows were fed corn silage TMR (periods 1 and 3) and Italian ryegrass silage TMR (period 2).

a,b Means with different letters significantly differ (p<0.05).

**Table 2:** Effects of feeding periods on feed intake, milk yield, and milk composition in dairy cows^1)^

Item	Period -1	Period -2	Period -3	SEM
DMI (kg/d)				
TMR	19.5±0.56	19.7±3.72	20.1±0.51	2.33
Concentrate	4.88±0.15	4.75±0.13	4.62±0.11	0.26
Milk yield (kg/d)	28.8±1.36	29.2±1.40	27.3±1.57	1.45
Milk composition (%)				
Fat	4.03±0.08	4.14±0.09	4.09±0.07	0.34
Protein	3.48±0.05	3.45±0.05	3.56±0.05	0.136
Lactose	4.57±0.03	4.57±0.04	4.54±0.04	0.076
SNF	8.97±0.06	9.03±0.05	9.02±0.06	0.183
SCC (×1,000/mL)	138±45	145±44	148±39	12.7

SEM, standard error of means; DMI, dry matter intake; TMR, total mixed ration; SNF, solids-not-fat; d, day; SCC, somatic cell count.

1) Cows were fed corn silage TMR (periods 1 and 3) and Italian ryegrass silage TMR (period 2).

**Table 3:** Effects of feeding periods on fatty acid composition (% of total fatty acid) in milk of dairy cows

Fatty acids	Period-1^1)^	Period-2^1)^	Period-3^1)^	SEM
C8	0.69±0.09	0.64±0.06	0.89±0.12	0.079
C10	3.06±0.13	3.05±0.12	3.36±0.15	0.093
C12	4.60±0.13	4.63±0.16	4.91±0.15	0.073
C14	13.8±0.23	13.5±0.25	13.9±0.23	0.162
C14:1	2.77±0.64	2.9±0.11	2.87±0.09	0.064
C15	1.27±0.05	1.32±0.05	1.29±0.05	0.029
C16	34.7±0.73	34.0±0.70	34.2±0.72	0.411
C16:1	2.32±0.11	2.36±0.07	2.23±0.08	0.057
C17	0.52±0.02	0.53±0.02	0.50±0.02	0.016
C18	5.89±0.84	4.61±0.97	4.18±0.90	0.764
*trans*-11 C18:1	3.42±0.21	6.10±0.07	5.72±0.20	1.315
*cis*-9 C18:1	24.4±0.72	24.5±0.67	23.8±0.63	0.31
*cis*-9, 12 C18:2	0.94±0.03	0.92±0.03	0.96±0.03	0.023
*cis*-9, 12, 15 C18:3	0.59±0.03	0.67±0.03	0.67±0.04	0.035

SEM, standard error of means.

1) Cows were fed corn silage total mixed ration (periods 1 and 3) and Italian ryegrass silage total mixed ration (period 2).

**Table 4:** Effects of feeding periods on plasma metabolite concentrations in dairy cows

Item	Period-1^1)^	Period-2^1)^	Period-3^1)^	SEM
Glucose (mmol/L)	3.98±0.07	3.74±0.06	3.99±0.07	0.063
TG (μmol/L)	63.9±3.08	73.8±4.45	70.8±5.45	4.14
T-CHO (mmol/L)	5.73±0.28	5.81±0.28	5.41±0.31	0.257
NEFA (μEq/L)	110.1±5.95	100.3±4.71	112.5±4.92	5.1
BUN (mmol/L)	1.96±0.10^b^	2.72±0.10^a^	1.98±0.08^b^	0.109

SEM, standard error of means; TG, triglyceride; T-GHO, total cholesterol; NEFA, non-esterified fatty acids; BUN, blood urea nitrogen.

1) Cows were fed corn silage TMR (periods 1 and 3) and Italian ryegrass silage TMR (period 2).

a,b Means with different letters significantly differ (p<0.05).

**Table 5:** Effects of feeding periods on milk and feces phytanic acid in dairy cows

Item	n	Period-1^1)^	Period-2^1)^	Period-3^1)^	SEM
Phytanic acid in milk (mg/kg)	17	9.30±0.38^b^	13.9±0.84^a^	8.80±0.38^b^	0.378
Phytanic acid secretion in milk (mg/d)	17	269.8±28^b^	415.6±34^a^	247.5±30^b^	14.04
Phytanic acid in faeces (mg/kg DM)	4	1.15±0.04^b^	1.65±0.04^a^	1.17±0.03^b^	0.022
Phytol in faeces (g/kg DM)	4	0.515±0.04	0.455±0.02	0.492±0.01	0.035

SEM, standard error of means; DM, dry matter; d, day.

1) Cows were fed corn silage total mixed ration (periods 1 and 3) and Italian ryegrass silage total mixed ration (period 2).

a,b Means with different letters significantly differ (p<0.05).
